# Comparative Effectiveness and Safety of Adalimumab, Secukinumab, and Upadacitinib in Psoriatic Arthritis: A Prospective Cohort Study Based on PARWCH Cohort

**DOI:** 10.1111/1346-8138.17906

**Published:** 2025-08-17

**Authors:** Yiyi Wang, Jingya Gao, Furong Li, Luyuan Li, Yue Xiao, Hongxiang Hu, Xiya Peng, Min Yang, Dan Hao, Wei Yan, Dengmei Xia, Wei Li

**Affiliations:** ^1^ Department of Dermatology, Rare Diseases Center West China Hospital, Sichuan University Chengdu China; ^2^ The First School of Clinical Medicine Southern Medical University Guangzhou Guangdong China; ^3^ Department of Rheumatology West China Hospital, Sichuan University Chengdu China; ^4^ Department of Dermatology West China Second University Hospital, Sichuan University Chengdu China

**Keywords:** adalimumab, psoriasis, psoriatic arthritis, secukinumab, targeted therapies, upadacitinib

## Abstract

Psoriatic arthritis (PsA) is a chronic inflammatory disease, with prevalence among psoriasis patients ranging from 6% to 42% across populations. Although targeted therapies such as adalimumab (ADA), secukinumab (SEC), and upadacitinib (UPA) have demonstrated efficacy in randomized controlled trials, real‐world head‐to‐head comparisons remain limited. This study aimed to compare the real‐world effectiveness and safety of ADA, SEC, and UPA in PsA patients. We conducted a prospective cohort study using data from the PARWCH database. PsA patients treated with ADA, SEC, or UPA were included and followed at baseline, Week 4, Week 12, and Week 24. Skin responses were evaluated using PASI75/90. Joint outcomes—including peripheral and axial arthritis—were assessed with ACR, PsARC, and ASAS criteria. Patient‐reported pain, disease activity, and HAQ scores were also recorded. Adverse events (AEs) were monitored throughout treatment. MMRM and GLMM were used to analyze continuous and binary outcomes, respectively. A total of 187 PsA patients were included (SEC: 78; ADA: 66; UPA: 43). All three agents demonstrated comparable effectiveness in improving peripheral joint symptoms (ACR20: SEC vs. ADA, Coef = −0.29, *p* = 0.62; UPA vs. ADA, Coef = −0.29, *p* = 0.66) and axial involvement (ASAS20: SEC vs. ADA, Coef = −0.04, *p* = 0.81; UPA vs. ADA, Coef = −1.05, *p* = 0.23). UPA and SEC showed significantly greater effectiveness than ADA in improving skin lesions (PASI90: SEC vs. ADA, Coef = 1.84, *p* = 0.006; UPA vs. ADA, Coef = 1.53, *p* = 0.04). However, ADA was more effective in relieving pain compared to both UPA (Coef = 2.43, *p* < 0.001) and SEC (Coef = 1.21, *p* = 0.02). Over 24 weeks, 85 AEs were reported by 48 patients, with fatigue, rash, upper respiratory tract infection, and pruritus being the most common. No serious AEs occurred. In conclusion, UPA and SEC demonstrated balanced effectiveness across skin and joint domains, while ADA offered superior pain relief. These findings support personalized treatment strategies tailored to the clinical features of PsA patients.

## 1 Introduction

Psoriatic arthritis (PsA) is a chronic, inflammatory musculoskeletal disease characterized by considerable clinical heterogeneity, encompassing six key domains: peripheral arthritis, axial spondylitis, enthesitis, dactylitis, skin lesions, and nail psoriasis [1, 2]. Epidemiological studies have reported that the prevalence of PsA among patients with psoriasis ranges from 6% to 42% across different populations [3]. In China, the prevalence of PsA in the psoriatic population is estimated at around 10.6% [4]. PsA often leads to functional impairment and a significant decline in quality of life, imposing a substantial burden on affected individuals. Despite ongoing advances, identifying effective and safe treatment strategies for PsA remains a critical and persistent challenge in clinical practice [5].

Conventional treatment for PsA has relied on nonsteroidal anti‐inflammatory drugs and conventional disease‐modifying antirheumatic drugs, which primarily provide symptom relief but show limited ability to modify or reduce disease progression [6, 7]. The introduction of tumor necrosis factor inhibitors, such as adalimumab (ADA), marked a significant advancement in PsA management by directly targeting the proinflammatory cytokine TNF‐α [6]. More recently, secukinumab (SEC), a fully human monoclonal IgG1 antibody that inhibits IL‐17A, has been approved for the treatment of PsA to further control disease activity and progression [8]. Both agents have shown substantial effectiveness and are recommended by the Group for Research and Assessment of Psoriasis and Psoriatic Arthritis as first‐line biologic therapies for managing the six key domains of PsA [9].

However, the pathogenesis of PsA is complex, involving a wide range of immune cell types and signaling pathways [10]. Consequently, therapies targeting a single cytokine may not provide sufficient effectiveness for all patients. In recent years, small‐molecule drugs—particularly Janus kinase (JAK) inhibitors—have emerged as promising therapeutic options [11, 12, 13, 14, 15]. By blocking the JAK–STAT signaling pathway, which plays a central role in the activation and differentiation of various immune cells, JAK inhibitors exert broad immunomodulatory effects and have shown potential across multiple immune‐mediated inflammatory diseases [16]. Upadacitinib (UPA), a selective JAK1 inhibitor, has demonstrated both efficacy and safety and has been approved for PsA treatment. In a head‐to‐head randomized controlled trial, UPA was shown to be noninferior to ADA in terms of efficacy, with a favorable long‐term safety profile [17, 18]. These findings position UPA as a promising small‐molecule option for PsA management. Nevertheless, real‐world evidence on UPA, especially in Chinese populations, remains limited. Moreover, direct comparisons between UPA and key biologics such as SEC and ADA are lacking in real‐world settings.

The objective of this study is to enhance the real‐world evidence for UPA based on data from a Chinese PsA cohort and to compare the effectiveness and safety of UPA with SEC and ADA.

## 2 Materials and Methods

### 2.1 Patients and Study Design

All patients' data were obtained from the PARWCH [19], a prospective cohort that has been collecting clinical data of patients with PsA who were treated at the Department of Dermatology, West China Hospital since March 2020. For this study, we included adult patients diagnosed with PsA between March 2020 and December 2024 who received treatment with either secukinumab (300 mg subcutaneous injection at baseline, followed by doses at Weeks 1, 2, 3, and 4, and subsequently every 4 weeks), adalimumab (40 mg subcutaneous injection every 2 weeks), or upadacitinib (15 mg orally once daily). PsA diagnoses were established according to the Classification Criteria for Psoriatic Arthritis [20]. Exclusion criteria were defined based on protocols previously established at our center [21]. Briefly, patients with coexisting forms of arthritis, including rheumatoid arthritis, osteoarthritis, gouty arthritis, and ankylosing spondylitis, were excluded. Additionally, individuals with hematological disorders, malignant tumors, severe infectious diseases, or significant hepatic, renal, or other visceral organ dysfunction were not eligible for inclusion. Pregnant or lactating women were also excluded from the study.

This study was approved by the Ethics Committee of West China Hospital, Sichuan University (approval number: 2020 (321)). Written informed consent was obtained from all participants prior to their participation in the study.

### 2.2 Clinical Assessment

A comprehensive clinical assessment was performed for each patient at baseline, encompassing five key domains: Demographic characteristics, disease‐related histories, personal and family histories, prior treatments, and physical examinations. The specific parameters evaluated are detailed in Table 1. All effectiveness endpoints were assessed at Week 4, Week 12, and Week 24. Treatment responses were assessed based on the proportions of patients achieving ≥ 20%, ≥ 50%, and ≥ 70% improvement according to the American College of Rheumatology criteria (ACR20/50/70); ≥ 20%, ≥ 40%, and ≥ 70% improvement in the Assessment of SpondyloArthritis International Society criteria (ASAS20/40/70); ≥ 75% and ≥ 90% improvement in the Psoriasis Area and Severity Index (PASI75/90); as well as the proportion of patients meeting the Psoriatic Arthritis Response Criteria (PsARC) and Minimal Disease Activity (MDA). Additionally, changes from baseline were recorded for several clinical measures, including the pain visual analog scale (VAS), disease activity VAS, Leeds Enthesitis Index (LEI), back pain VAS, global VAS, and Health Assessment Questionnaire (HAQ). Safety data through 24 weeks were summarized by treatment groups for all included patients.

**TABLE 1:** Baseline information of patients in SEC, ADA, and UPA groups.

Variables	Total (*n* = 187)	SEC (*n* = 78)	ADA (*n* = 66)	UPA (*n* = 43)	*p*
Demographic information					
Sex, male	118 (63.10)	51 (65.38)	46 (69.70)	21 (48.84)	0.07
Age, years	42.24 (12.39)	43.13 (12.59)	42.30 (12.71)	40.53 (11.61)	0.52
BMI, kg/m^2^	23.98 (3.69)	23.86 (3.25)	24.14 (4.23)	23.93 (3.61)	0.91
Onset age					
Psoriasis onset age, years	27.91 (12.27)	27.71 (11.28)	26.35 (12.09)	30.65 (13.97)	0.26
PsA onset age, years	37.46 (12.22)	38.32 (13.13)	37.29 (11.39)	36.17 (11.90)	0.66
Onset order					
PsO earlier than arthritis	160 (85.56)	70 (89.74)	57 (86.36)	33 (76.74)	
Arthritis earlier than PsO	17 (9.09)	3 (3.85)	5 (7.58)	9 (20.93)	0.05
Simultaneous onset	10 (5.35)	5 (6.41)	4 (6.06)	1 (2.33)	
Comorbidities					
Hypertension	27 (14.44)	12 (15.38)	12 (18.18)	3 (6.98)	0.25
Type 2 diabetes	11 (5.88)	5 (6.41)	4 (6.06)	2 (4.65)	1.00
Cardiovascular disease	3 (1.60)	2 (2.56)	1 (1.52)	0 (0.00)	0.80
Fatty liver	70 (37.43)	30 (38.46)	23 (34.85)	17 (39.53)	0.88
Family histories					
Psoriasis	44 (23.53)	17 (21.79)	18 (27.27)	9 (20.93)	0.69
PsA	9 (4.81)	4 (5.13)	4 (6.06)	1 (2.33)	0.82
Personal histories					
Smoking	69 (36.90)	30 (38.46)	27 (40.91)	12 (27.91)	0.39
Alcohol	69 (36.90)	23 (29.49)	25 (37.88)	21 (48.84)	0.11
Surgery	70 (37.43)	30 (38.46)	24 (36.36)	16 (37.21)	0.98
Trauma	33 (17.65)	16 (20.51)	14 (21.21)	3 (6.98)	0.10
Previous treatment					
Biological history	44 (23.53)	21 (26.92)	17 (25.76)	6 (13.95)	0.23
IL‐17i	17 (9.09)	11 (14.10)	5 (7.58)	1 (2.33)	0.10
TNFi	39 (20.86)	20 (25.64)	14 (21.21)	5 (11.63)	0.19
IL‐12/23i	2 (1.07)	1 (1.28)	1 (1.52)	0 (0.00)	1.00
Methotrexate	88 (47.06)	36 (46.15)	33 (50.00)	19 (44.19)	0.83
Acitretin	54 (28.88)	23 (29.49)	23 (34.85)	8 (18.60)	0.19
Cyclosporine	2 (1.07)	1 (1.28)	1 (1.52)	0 (0.00)	1.00
TCM	38 (20.32)	17 (21.79)	17 (25.76)	4 (9.30)	0.10
Phototherapy	44 (23.53)	24 (30.77)	17 (25.76)	3 (6.98)	0.01
NSAIDs	47 (25.13)	20 (25.64)	10 (15.15)	17 (39.53)	0.02
Topical therapy	25 (13.37)	8 (10.26)	6 (9.09)	11 (25.58)	0.04
Subtype					
Axial	16 (8.56)	10 (12.82)	2 (3.03)	4 (9.30)	
Peripheral	60 (32.09)	24 (30.77)	20 (30.30)	16 (37.21)	0.22
Mixed	111 (59.35)	44 (56.41)	44 (66.67)	23 (53.49)	
Nail involvement					
Nail bed	12 (6.42)	7 (8.97)	3 (4.55)	2 (4.65)	
Nail matrix	42 (22.46)	13 (16.67)	19 (28.78)	10 (23.26)	0.64
Both	65 (34.76)	27 (34.62)	23 (34.85)	15 (34.88)	
None	68 (36.36)	31 (39.74)	21 (31.82)	16 (37.21)	
Psoriasis severity					
PASI	5.85 (8.43)	7.22 (8.44)	7.00 (9.89)	1.60 (2.98)	< 0.001
HAQ	0.28 (0.59)	0.28 (0.63)	0.29 (0.55)	0.27 (0.59)	0.99
Global VAS	5.35 (2.49)	5.42 (2.58)	5.80 (2.34)	4.55 (2.40)	0.03
Peripheral involvement^a^					
TJC	8.94 (13.32)	8.47 (13.08)	11.00 (14.60)	6.36 (11.15)	0.20
SJC	3.63 (7.06)	2.66 (5.03)	4.62 (6.73)	3.69 (10.00)	0.17
Pain VAS	4.83 (2.80)	4.62 (2.75)	5.48 (2.76)	4.13 (2.78)	0.04
Disease activity VAS‐patient	5.27 (2.89)	5.57 (2.80)	5.84 (2.52)	3.79 (3.19)	0.004
Disease activity VAS‐clinician	4.89 (2.77)	5.15 (2.57)	5.13 (2.65)	4.03 (3.17)	0.14
LEI	0.44 (1.11)	0.38 (1.12)	0.62 (1.25)	0.23 (0.74)	0.14
Axial involvement^b^					
BASFI	1.68 (2.26)	1.85 (2.48)	1.94 (2.31)	0.89 (1.52)	0.04
BASDAI	2.06 (2.54)	2.08 (2.52)	2.35 (2.95)	1.54 (1.67)	0.29
Back pain VAS	3.34 (3.02)	2.90 (2.86)	3.91 (3.12)	3.26 (3.12)	0.25

*Note:* Continuous variables are presented as mean (SD), while categorial variables are presented as count (%).

Abbreviations: ADA: Adalimumab; BASDAI: Bath ankylosing spondylitis disease activity index; BASFI: Bath ankylosing spondylitis functional index; BMI: Body mass index; HAQ: Health assessment questionnaire; LEI: Leeds enthesitis index; NSAIDs: Nonsteroidal anti‐inflammatory drugs; PASI: Psoriasis area and severity index; PsA: Psoriatic arthritis; PsO: psoriasis with only skin involvement; SEC: Secukinumab; SJC: Swollen joint count; TCM: Traditional Chinese medicine; TJC: Tender joint count; UPA: Upadacitinib; VAS: Visual analog scale.

^a^ Sixty‐eight patients in the SEC group, 64 patients in the ADA group, and 41 patients in the UPA group have peripheral arthritis.

^b^ Fifty‐four patients in the SEC group, 46 patients in the ADA group, and 27 patients in the UPA group have axial arthritis.

### 2.3 Statistical Analysis

Baseline continuous variables were presented as mean (standard deviation) and compared among the three groups using a one‐way ANOVA test. Categorical variables were expressed as counts and percentages and compared using the Chi‐square test or Fisher's exact test. Effectiveness analyses included all patients, regardless of treatment discontinuation or loss to follow‐up. For continuous endpoints (changes from baseline in pain VAS, disease activity VAS, LEI, back pain VAS, global VAS, and HAQ), a mixed model for repeated measures (MMRM) was applied to observed data. The model included treatment group, visit, and treatment‐by‐visit interaction as fixed effects, with age, body mass index (BMI), baseline PASI, and biological history included as covariates. Missing data were handled under the missing‐at‐random assumption via MMRM. Binary endpoints (proportion of patients achieving ACR20/50/70, ASAS20/40/70, PASI75/90, PsARC, and MDA) were analyzed using generalized linear mixed models (GLMM) with the same fixed effects and covariates as in the MMRM. Results were reported as estimates and *p* values, with *p* < 0.05 considered statistically significant. Furthermore, outcomes were also compared among the three groups at each follow‐up time point. For binary outcomes, logistic regression was used to calculate *p* values, adjusting for age, BMI, baseline PASI score, and biological treatment history, consistent with the MMRM model. For continuous outcomes, linear regression was applied with the same covariate adjustments. Safety data were summarized as the number and percentage of patients experiencing adverse events. Analyses and plotting were conducted using R v4.3.0.

## 3 Results

### 3.1 Patients

A total of 187 patients (SEC, *n* = 78; ADA, *n* = 66; UPA, *n* = 43) were included in the study. Among them, 162 (86.63%) completed the full 24‐week follow‐up, while 25 (13.37%) had missing data at various time points. Most patients in this cohort were male (118, 63.10%), with a mean baseline age of 42.24 years (SD 12.39). Regarding disease subtype, 171 (91.44%) patients had peripheral or mixed PsA, whereas only 16 (8.56%) exhibited exclusive axial involvement (Table 1).

Among all baseline variables, age, sex, BMI, PASI, disease activity VAS, global VAS, and history of biologic use were considered potential confounders due to their possible associations with treatment response to biologics in PsA [22, 23, 24]. After assessing for multicollinearity through correlation analysis, collinear variables were excluded (Figure S1). Ultimately, age, BMI, baseline PASI, and biological history were included as covariates in the MMRM, GLMM, logistic regression, and linear regression models to adjust for potential confounding effects.

### 3.2 Effectiveness: Binary Outcomes

**FIGURE 1:**
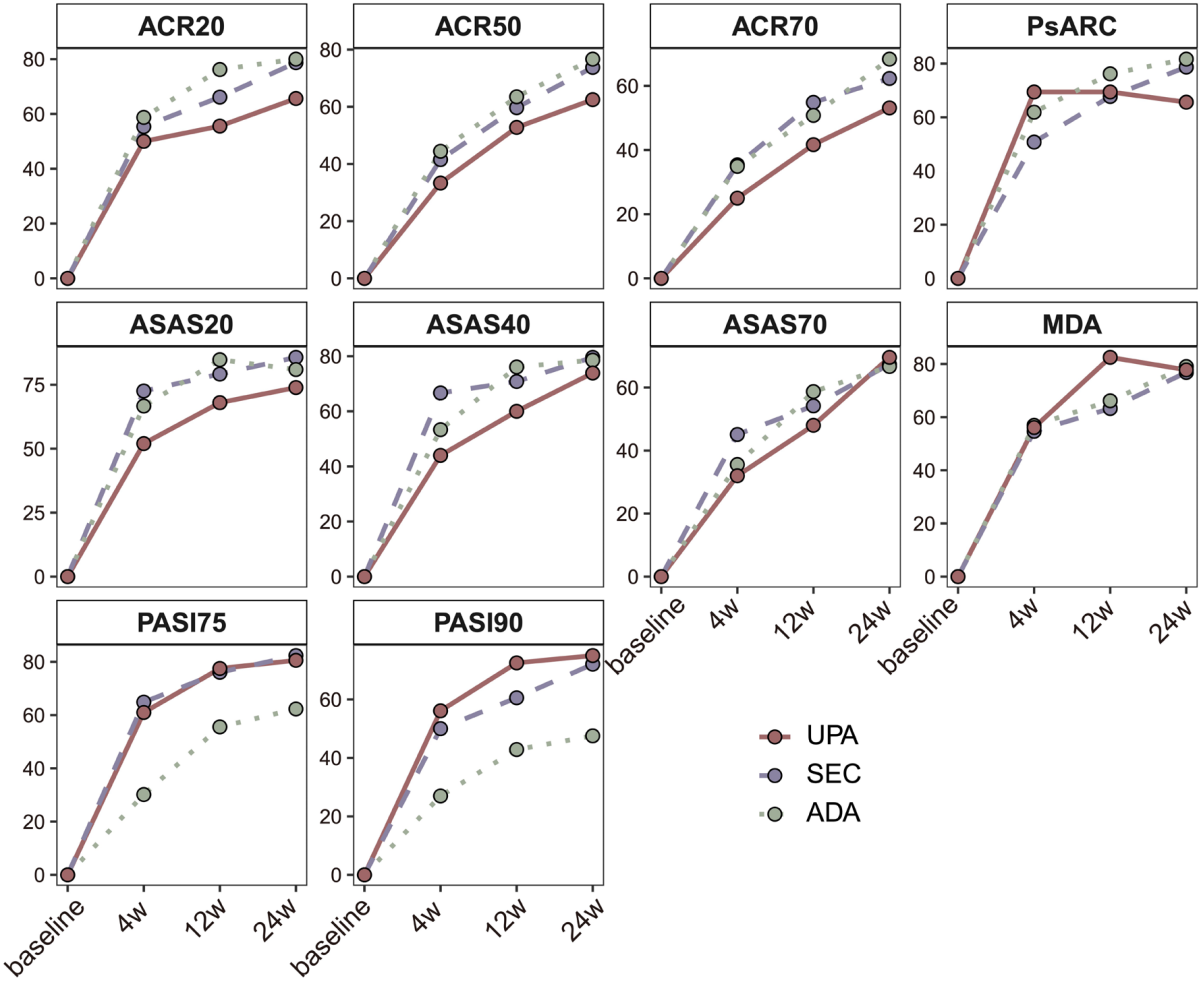
Changes of binary outcomes up to Week 24. The binary outcomes presented as response rates were presented at baseline, Weeks 4, 12, and 24. Abbreviations: ACR: American College of Rheumatology; ADA: adalimumab; ASAS: Assessment of SpondyloArthritis International Society; MDA: Minimal Disease Activity. PASI: Psoriasis Area and Severity Index; PsARC: Psoriatic Arthritis Response Criteria; SEC: secukinumab; UPA: Upadacitinib.

The proportions of patients achieving ACR20 increased over time, with rates of 55.49%, 67.70%, and 76.47% at Weeks 4, 12, and 24, respectively (Figure 1). No statistically significant differences in ACR20 responses were observed among the three treatment groups (GLMM: SEC vs. ADA: Coef = −0.29, *p* = 0.62; UPA vs. ADA: Coef = −0.29, *p* = 0.66; Table 2). Consistently, logistic regression analyses further confirmed that the ACR20 response rates were comparable across the three treatment groups at Weeks 4, 12, and 24 (Tables S1–S3). Similar trends were seen for ACR50 and ACR70 responses, with no significant intergroup differences (Figure 1, Table 2, Tables S1–S3). Among patients with axial PsA, although the UPA group showed slightly lower ASAS20/40/70 response rates at each time point compared to the SEC and ADA groups (Figure 1), no statistically significant differences were observed among the three treatment groups (Table 2, Tables S1–S3). Additionally, composite endpoints such as the PsARC (GLMM: SEC vs. ADA: Coef = −0.75, *p* = 0.14; UPA vs. ADA: Coef = 0.72, *p* = 0.24) and MDA response (GLMM: SEC vs. ADA: Coef = −0.28, *p* = 0.66; UPA vs. ADA: Coef = −0.46, *p* = 0.52) did not show significant differences among the three treatment groups. In terms of skin involvement, PASI75 (GLMM: SEC vs. ADA: Coef = 2.82, *p* < 0.001; UPA vs. ADA: Coef = 2.10, *p* = 0.008) and PASI90 responses (GLMM: SEC vs. ADA: Coef = 1.84, *p* = 0.006; UPA vs. ADA: Coef = 1.53, *p* = 0.04) were significantly higher in patients treated with SEC and UPA compared to those receiving ADA.

**TABLE 2:** Results of generalized linear mixed models.

Responses	SEC vs. ADA		UPA vs. ADA	
	Coef	*p*	Coef	*p*
ACR20	−0.29	0.62	−0.29	0.66
ACR50	−0.24	0.68	−0.25	0.71
ACR70	0.11	0.87	−0.59	0.42
PsARC	−0.75	0.14	0.72	0.24
ASAS20	−0.04	0.81	−1.05	0.23
ASAS40	0.57	0.53	−0.75	0.46
ASAS70	0.59	0.50	−0.60	0.57
PASI75	2.82	**< 0.001**	2.10	**0.008**
PASI90	1.84	**0.006**	1.53	**0.04**
MDA	−0.28	0.66	−0.46	0.52

*Note:* Adjusted by age, BMI, baseline PASI score, and biological history.

Abbreviations: ACR: American College of Rheumatology; ADA: Adalimumab; ASAS: Assessment of SpondyloArthritis International Society; MDA: Minimal Disease Activity. PASI: Psoriasis Area and Severity Index; PsARC: Psoriatic Arthritis Response Criteria; SEC: Secukinumab; UPA: Upadacitinib.

### 3.3 Effectiveness: Continuous Outcomes

**FIGURE 2:**
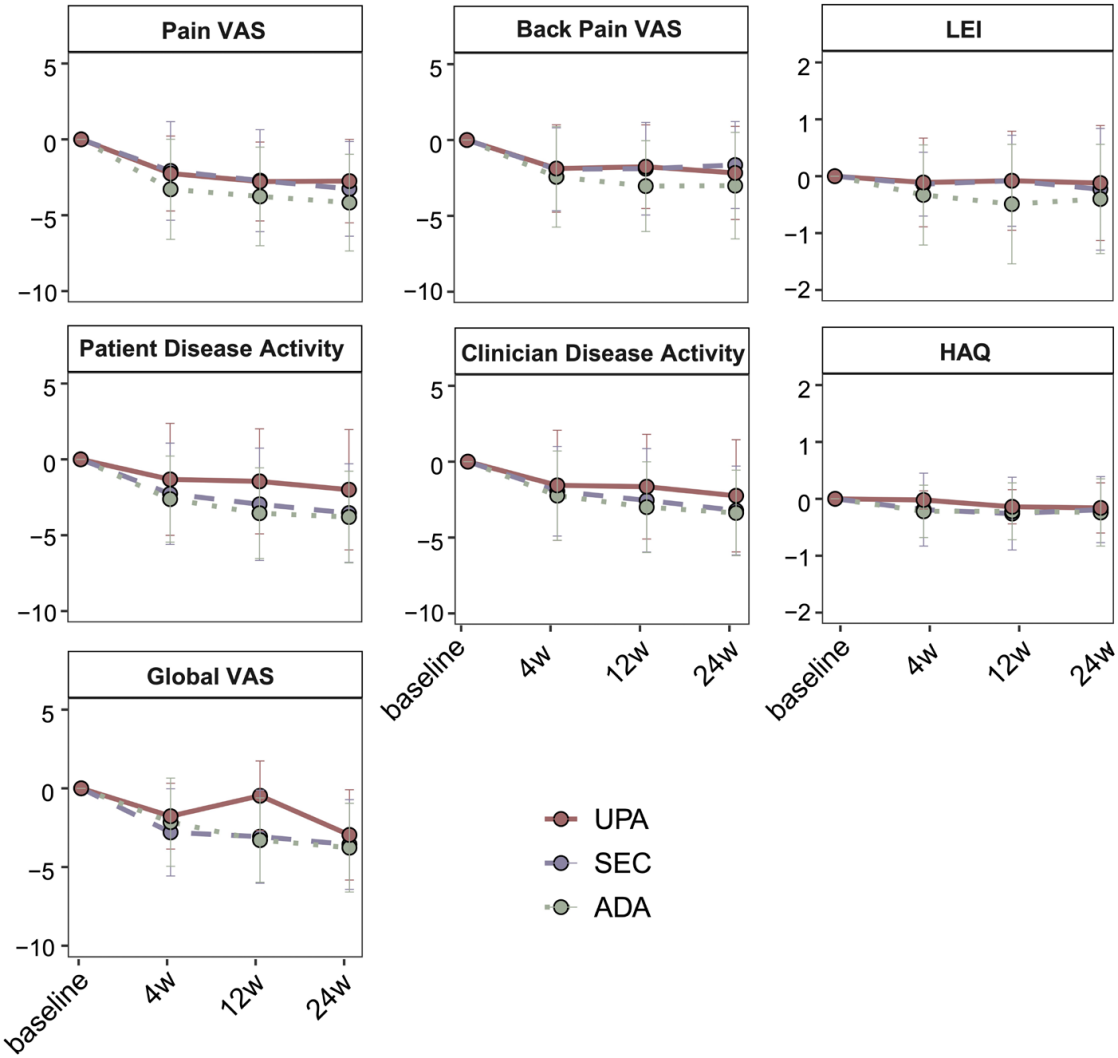
Changes of continuous outcomes up to Week 24. Continuous outcomes, shown as changes from baseline, were displayed at baseline and follow‐up visits (Weeks 4, 12, and 24). Data points represent mean values, and error bars denote standard deviations (mean ± SD). Abbreviations: ADA: Adalimumab; HAQ: Health assessment questionnaire; LEI: Leeds enthesitis index; SEC: Secukinumab; UPA: Upadacitinib; VAS: Visual analog scale.

As shown in Table 3 and Figure 2, the most pronounced improvement was observed in pain VAS, with ADA providing significantly greater pain relief than both SEC (MMRM: Coef = 1.21, *p* = 0.02) and UPA (MMRM: Coef = 2.43, *p* < 0.001). Similarly, in patients with axial PsA, ADA demonstrated superior effectiveness over UPA in alleviating back pain (MMRM: Coef = 1.39, *p* = 0.02). Furthermore, HAQ improvement appeared to be slightly greater with SEC, followed by ADA, and then UPA (MMRM: SEC vs. ADA: Coef = −0.13, *p* = 0.06; UPA vs. ADA: Coef = 0.13, *p* = 0.05). In time‐point‐specific analyses, ADA appeared to be more effective in alleviating enthesitis at Week 12, showing a greater reduction in LEI scores (−0.49 ± 1.05) compared to SEC (−0.08 ± 0.80, *p* = 0.02) and UPA (0.08 ± 0.87, *p* = 0.01) (Table S2, Figure 2). Meanwhile, UPA demonstrated relatively less improvement in patient‐reported disease activity compared with SEC and ADA at both Weeks 12 and 24 (Tables S2 and S3, Figure 2).

**TABLE 3:** Results of mixed effects model for repeated measures.

Change of variables	SEC vs. ADA		UPA vs. ADA	
	Coef	*p*	Coef	*p*
Pain VAS	1.21	**0.02**	2.43	**< 0.001**
Disease activity—patients	0.29	0.59	0.66	0.26
Disease activity—clinician	0.31	0.56	0.30	0.59
LEI	0.18	0.22	0.17	0.28
Back pain VAS	0.13	0.82	1.39	**0.02**
Global VAS	−0.47	0.28	0.65	0.17
HAQ	−0.13	0.06	0.13	0.05

*Note:* Adjusted by age, BMI, baseline PASI score, and biological history.

Abbreviations: ADA: Adalimumab; BMI: body mass index; HAQ: Health assessment questionnaire; LEI: Leeds enthesitis index; SEC: Secukinumab; UPA: Upadacitinib; VAS: Visual analog scale.

## 4 Safety

The safety profiles of the three treatments included reported events of interest to date, with 48 patients experiencing a total of 85 adverse events (AEs) during the 24‐week period. Fatigue was the most common AE (12, 6.42%), followed by rash (11, 5.88%), upper respiratory tract infection (9, 4.81%), and pruritus (9, 4.81%) (Table 4). No serious adverse events (SAEs) were reported during the 24‐week period (Table 4). Notably, no cases of venous thromboembolism, major adverse cardiovascular events, or malignancies were reported in the UPA group.

**TABLE 4:** Adverse events reported within the 24‐week study period.

Type of adverse event	Total (*n* = 187)	SEC (*n* = 78)	ADA (*n* = 66)	UPA (*n* = 43)
AE	48 (25.67)	20 (25.64)	26 (39.39)	2 (4.65)
SAE	0 (0.00)	0 (0.00)	0 (0.00)	0 (0.00)
Common AEs				
Fatigue	12 (6.42)	3 (3.85)	9 (13.64)	0 (0.00)
Rash	11 (5.88)	6 (7.69)	4 (6.06)	1 (2.33)
Upper respiratory tract infection	9 (4.81)	0 (0.00)	7 (10.61)	2 (4.65)
Pruritus	9 (4.81)	1 (1.28)	8 (12.12)	0 (0.00)
Pharyngitis	8 (4.28)	4 (5.13)	3 (4.55)	1 (2.33)
Arthralgia	8 (4.28)	8 (10.26)	0 (0.00)	0 (0.00)
Injection site reaction	7 (3.74)	1 (1.28)	6 (9.09)	0 (0.00)
Tinea pedis	5 (2.67)	0 (0.00)	5 (7.58)	0 (0.00)
Rhinitis	4 (2.14)	4 (5.13)	0 (0.00)	0 (0.00)
Insomnia	4 (2.14)	2 (2.56)	2 (3.03)	0 (0.00)
Conjunctivitis	3 (1.59)	0 (0.00)	3 (4.55)	0 (0.00)
Diarrhea	2 (1.60)	2 (2.56)	0 (0.00)	0 (0.00)
Tonsillitis	1 (0.53)	1 (1.28)	0 (0.00)	0 (0.00)
Others	2 (1.60)	0 (0.00)	2 (3.03)	0 (0.00)

Abbreviations: ADA, Adalimumab; AE, Adverse event; SAE, Serious adverse event; SEC, Secukinumab; UPA, Upadacitinib.

## 5 Discussion

This prospective cohort study provides a comprehensive real‐world comparison of ADA, SEC, and UPA in Chinese patients with PsA, evaluating both effectiveness and safety profiles. Our findings demonstrate comparable joint symptom improvement across all three therapies, reinforcing their established roles in PsA management. However, divergent effects on skin clearance and joint pain reduction were observed, highlighting potential therapeutic nuances that may guide personalized treatment decisions. Notably, all three agents exhibited acceptable safety profiles in real‐world practice, supporting their continued use in this population.

Firstly, we observed that the three treatments exhibit comparable effectiveness in joint symptom improvement, consistent with previous pivotal randomized controlled trials (RCTs). The SELECT‐PsA1 trial directly compared UPA and ADA, revealing similar ACR20 response rates at Week 12 [17], with UPA maintaining sustained efficacy through 56 weeks of follow‐up [18]. Likewise, the EXCEED study—a head‐to‐head trial of SEC versus ADA in 853 PsA patients—found no significant difference in ACR20 response at Week 52 [25]. Notably, these RCTs exclusively enrolled peripheral PsA populations, leaving the question of axial involvement unaddressed. Our findings extend beyond these RCTs by demonstrating comparable effectiveness of UPA, SEC, and ADA in both peripheral and axial PsA—a critical observation given that up to 59.36% of patients exhibit mixed phenotypes. This phenotypic inclusivity in real‐world practice reinforces the broad applicability of these therapies across PsA manifestations.

However, nuanced differences emerged in psoriatic skin clearance and arthralgia improvement among the three therapies. Both SEC and UPA demonstrated superior effectiveness over ADA in cutaneous psoriasis improvement. SEC has historically shown dominant advantages in psoriatic lesion clearance, particularly when compared to ADA—a finding consistently replicated across multiple trials [25, 26]. While the SELECT‐PsA1 study [17] confirmed UPA's superiority over ADA in achieving PASI75 responses, direct comparisons between UPA and SEC for skin outcomes remain scarce in existing literature. Our real‐world data notably suggest that UPA possesses comparable effectiveness to SEC in psoriatic lesion improvement. Conversely, ADA demonstrated the most pronounced effect in alleviating arthralgia, which may be mechanistically explained by the pivotal role of TNF‐α in pain modulation. TNF‐α contributes to both inflammatory and neuropathic pain by directly sensitizing nociceptors and promoting the release of other proinflammatory cytokines, thereby amplifying peripheral and central pain signaling pathways [27].

During the 24‐week observation period, all three therapies exhibited safety profiles consistent with those reported in previous RCTs and real‐world studies [17, 25, 28, 29, 30], with no newly identified AEs. UPA was associated with the lowest overall incidence of AEs, indicating favorable short‐term tolerability. However, the limited duration of follow‐up restricts the ability to assess long‐term safety risks. Therefore, ongoing monitoring, risk stratification, and longer‐term real‐world studies are needed to fully characterize the long‐term safety profiles of these therapies.

Our study has several limitations that warrant consideration. First, as a single‐center prospective cohort with a limited sample size, the generalizability of our findings may be constrained, requiring validation through multicenter studies with more diverse populations. Second, it is worth noting that at baseline, there were substantial differences among the three treatment groups in PASI scores and patient‐reported disease activity VAS scores. Moreover, collinearity analysis revealed a strong positive correlation between these two variables. To minimize potential confounding, baseline PASI was included as an important covariate in all subsequent models—including MMRM, GLMM, logistic regression, and linear regression. Nevertheless, we acknowledge that some residual confounding may still be present and could not be fully controlled. Finally, the 24‐week observation period, while sufficient for initial effectiveness assessment, precludes evaluation of long‐term outcomes, including delayed safety signals and treatment durability. These limitations highlight the need for larger, longer term studies to confirm our real‐world findings.

In conclusion, we comprehensively validated the effectiveness and safety of UPA in this real‐world study of Chinese PsA patients. UPA demonstrated comparable effectiveness to SEC in psoriatic lesion improvement while showing comparable joint symptom relief to ADA, coupled with a favorable safety profile. As an oral small‐molecule agent, UPA offers notable convenience in clinical practice, positioning it as an excellent therapeutic option for PsA management. SEC maintained its established advantages in both cutaneous and articular outcomes, while ADA exhibited particularly robust effects on joint pain alleviation alongside its articular benefits. These findings provide valuable evidence for personalized treatment selection in PsA care, emphasizing the importance of considering dominant disease manifestations and administration preferences when choosing among these targeted therapies.

## Disclosure

The authors have nothing to report.

## Ethics Statement

This study was approved by the Ethics Committee of West China Hospital, Sichuan University (approval number: 2020(321)). Written informed consent was obtained from all participants prior to their participation in the study.

## Conflicts of Interest

The authors declare no conflicts of interest.

## Supporting information


**Figure S1:** Covariate correlation analysis. The pie charts in the figure represent correlation coefficients, and * indicates *p* values less than 0.05. BMI, Body Mass Index; PASI, Psoriasis Area and Severity Index; VAS, Visual Analog Scale.

**Table S1:** Treatment effectiveness and outcomes at Week 4.
**Table S2:**. Treatment effectiveness and outcomes at Week 12.
**Table S3:**. Treatment effectiveness and outcomes at Week 24.

## Data Availability

The data that support the findings of this study are available from the corresponding author upon reasonable request.
