# Supplementary figures and images for: Comparative Effectiveness and Safety of Adalimumab, Secukinumab, and Upadacitinib in Psoriatic Arthritis: A Prospective Cohort Study Based on PARWCH Cohort
Source: J Dermatol. 2025 Aug 17;52(10):1527–35. doi: 10.1111/1346-8138.17906 (PMC12530465; doi:10.1111/1346-8138.17906)

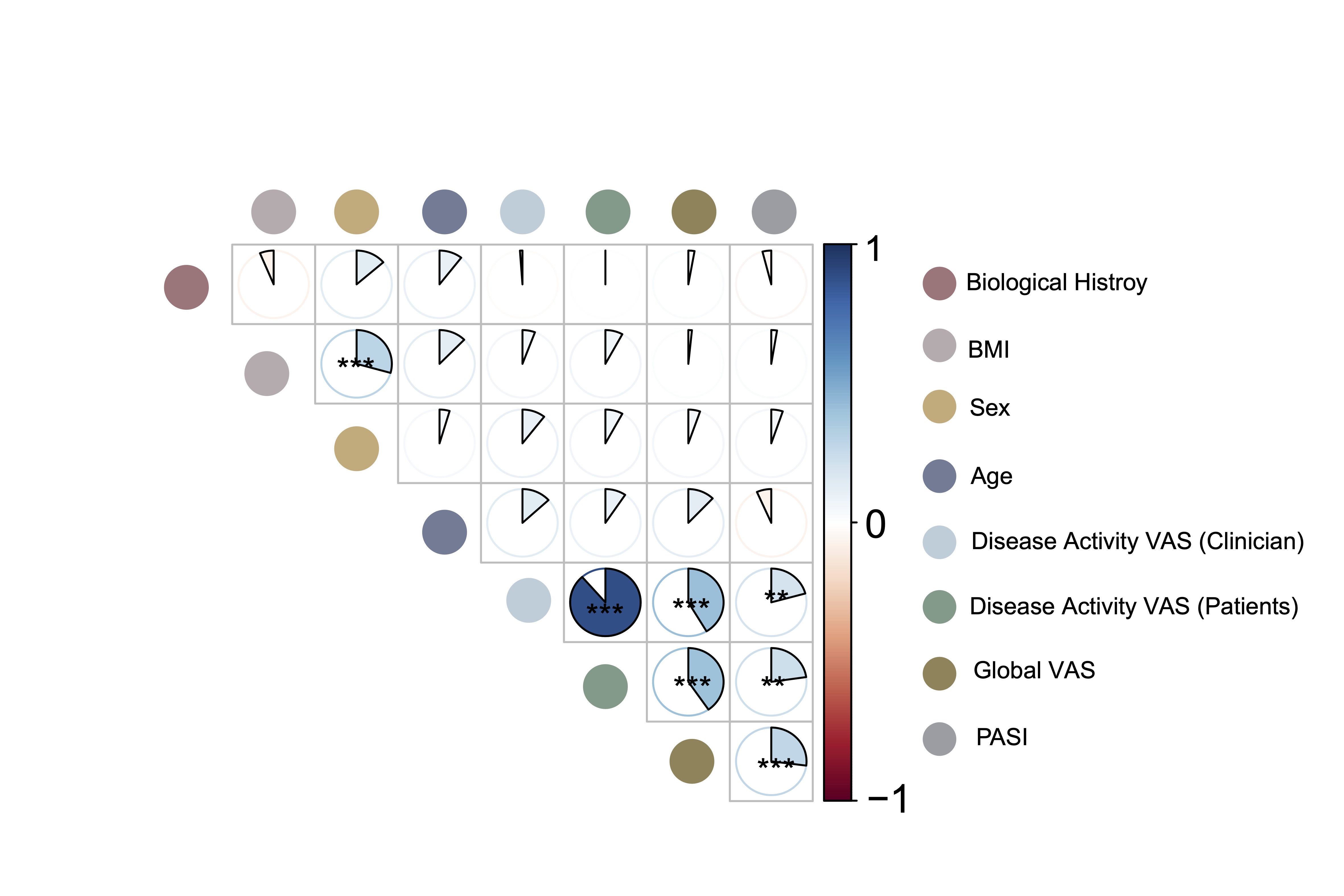

Supplement: Supplementary file 1 — Figure S1: Covariate correlation analysis. The pie charts in the figure represent correlation coefficients, and * indicates p values less than 0.05. BMI, Body Mass Index; PASI, Psoriasis Area and Severity Index; VAS, Visual Analog Scale. [file JDE-52-1527-s001.jpg]
