# Supplementary material for: Comparative Effectiveness and Safety of Adalimumab, Secukinumab, and Upadacitinib in Psoriatic Arthritis: A Prospective Cohort Study Based on PARWCH Cohort
Source: J Dermatol. 2025 Aug 17;52(10):1527–35. doi: 10.1111/1346-8138.17906 (PMC12530465; doi:10.1111/1346-8138.17906)
Supplement: Supplementary file 2 — Table S1: Treatment effectiveness and outcomes at Week 4. Table S2:. Treatment effectiveness and outcomes at Week 12. Table S3:. Treatment effectiveness and outcomes at Week 24. [file JDE-52-1527-s002.docx]

**Supplementary Table S1. Treatment effectiveness and outcomes at week 4.**

| **Variable** | **SEC** | **ADA** | **UPA** | ***P1*** | ***P2*** |
| --- | --- | --- | --- | --- | --- |
| **Peripheral arthritis** | n = 65 | n = 63 | n = 36 |  |  |
| ACR20 | 36 (55.38) | 37 (58.73) | 18 (50.00) | 0.80 | 0.40 |
| ACR50 | 27 (41.54) | 28 (44.44) | 12 (33.33) | 0.78 | 0.42 |
| ACR70 | 23 (35.38) | 22 (34.92) | 9 (25.00) | 0.85 | 0.32 |
| PsARC | 33 (50.77) | 39 (61.90) | 25 (69.44) | 0.20 | 0.31 |
| Change of pain VAS | -2.08 (3.25) | -3.29 (3.30) | -2.25 (2.47) | **0.03** | 0.16 |
| Change of LEI | -0.14 (0.56) | -0.33 (0.88) | -0.11 (0.78) | 0.13 | 0.10 |
|  |  |  |  |  |  |
| **Axial arthritis** | n = 51 | n = 45 | n = 25 |  |  |
| ASAS20 | 37 (72.55) | 30 (66.67) | 13 (52.00) | 0.64 | 0.59 |
| ASAS40 | 34 (66.67) | 24 (53.33) | 11 (44.00) | 0.18 | 0.51 |
| ASAS70 | 23 (45.10) | 16 (35.56) | 8 (32.00) | 0.34 | 0.61 |
| Change of back pain VAS | -1.93 (2.73) | -2.43 (3.31) | -1.88 (2.88) | 0.45 | 0.76 |
|  |  |  |  |  |  |
| **Skin** | n = 74 | n = 63 | n = 41 |  |  |
| PASI90 | 37 (50.00) | 17 (26.98) | 23 (56.10) | **0.006** | **0.04** |
| PASI75 | 48 (64.86) | 19 (30.16) | 25 (60.98) | **<0.001** | **0.01** |
|  |  |  |  |  |  |
| **Global evaluation** | n = 75 | n = 65 | n = 41 |  |  |
| MDA | 41 (54.67) | 37 (56.92) | 23 (56.10) | 0.96 | 0.59 |
| Change of HAQ | -0.19 (0.64) | -0.22 (0.46) | -0.02 (0.16) | 0.61 | 0.12 |
| Change of disease activity-patients | -2.27 (3.34) | -2.62 (2.84) | -1.32 (3.69) | 0.59 | 0.12 |
| Change of disease activity-clinician | -1.95 (2.95) | -2.24 (2.94) | -1.56 (3.63) | 0.62 | 0.45 |
| Change of global VAS | -2.80 (2.77) | -2.15 (2.80) | -1.77 (2.09) | 0.13 | 0.49 |

Continuous variables are presented as mean (SD), and categorical variables are presented as counts (%). For binary outcomes, comparisons between treatment groups were performed using multivariable logistic regression models, adjusting for age, BMI, baseline PASI score, and history of prior biologic treatment. For continuous outcomes, multivariable linear regression models were used with the same covariate adjustments. *P1* represents the p-value for the comparison between SEC and ADA, while *P2* represents the p-value for the comparison between UPA and ADA. SEC, Secukinumab; ADA, Adalimumab; UPA, Upadacitinib; ACR, American College of Rheumatology; PsARC, Psoriatic Arthritis Response Criteria; VAS, Visual Analog Scale; LEI, Leeds Enthesitis Index; ASAS, Assessment of SpondyloArthritis International Society; PASI, Psoriasis Area and Severity Index; MDA, Minimal Disease Activity; HAQ, Health Assessment Questionnaire.

**Supplementary Table S2. Treatment effectiveness and outcomes at week 12.**

| **Variable** | **SEC** | **ADA** | **UPA** | ***P1*** | ***P2*** |
| --- | --- | --- | --- | --- | --- |
| **Peripheral arthritis** | n = 62 | n = 63 | n = 36 |  |  |
| ACR20 | 41 (66.13) | 48 (76.19) | 20 (55.56) | 0.29 | **0.02** |
| ACR50 | 37 (59.68) | 40 (63.49) | 19 (52.78) | 0.77 | 0.23 |
| ACR70 | 34 (54.84) | 32 (50.79) | 15 (41.67) | 0.50 | 0.24 |
| PsARC | 42 (67.74) | 48 (76.19) | 25 (69.44) | 0.38 | 0.26 |
| Change of pain VAS | -2.72 (3.36) | -3.76 (3.25) | -2.78 (2.60) | 0.07 | 0.15 |
| Change of LEI | -0.08 (0.80) | -0.49 (1.05) | -0.08 (0.87) | **0.02** | **0.01** |
|  |  |  |  |  |  |
| **Axial arthritis** | n = 48 | n = 46 | n = 25 |  |  |
| ASAS20 | 38 (79.17) | 39 (84.78) | 17 (68.00) | 0.47 | 0.10 |
| ASAS40 | 34 (70.83) | 35 (76.09) | 15 (60.00) | 0.49 | 0.20 |
| ASAS70 | 26 (54.17) | 27 (58.70) | 12 (48.00) | 0.63 | 0.36 |
| Change of back pain VAS | -1.89 (3.05) | -3.04 (2.99) | -1.76 (2.76) | 0.06 | 0.22 |
|  |  |  |  |  |  |
| **Skin** | n = 71 | n = 63 | n = 40 |  |  |
| PASI90 | 43 (60.56) | 27 (42.86) | 29 (72.50) | **0.04** | **0.01** |
| PASI75 | 54 (76.06) | 35 (55.56) | 31 (77.50) | **0.01** | **0.03** |
|  |  |  |  |  |  |
| **Global evaluation** | n = 68 | n = 65 | n = 40 |  |  |
| MDA | 43 (63.24) | 43 (66.15) | 33 (82.50) | 0.96 | 0.14 |
| Change of HAQ | -0.26 (0.64) | -0.22 (0.50) | -0.14 (0.30) | 0.85 | 0.76 |
| Change of disease activity-patients | -2.96 (3.70) | -3.55 (2.99) | -1.45 (3.46) | 0.39 | **0.004** |
| Change of disease activity-clinician | -2.55 (3.41) | -3.00 (2.99) | -1.65 (3.45) | 0.50 | 0.06 |
| Change of global VAS | -3.07 (2.96) | -3.29 (2.68) | -0.47 (2.21) | 0.70 | **<0.001** |

Continuous variables are presented as mean (SD), and categorical variables are presented as counts (%). For binary outcomes, comparisons between treatment groups were performed using multivariable logistic regression models, adjusting for age, BMI, baseline PASI score, and history of prior biologic treatment. For continuous outcomes, multivariable linear regression models were used with the same covariate adjustments. *P1* represents the p-value for the comparison between SEC and ADA, while *P2* represents the p-value for the comparison between UPA and ADA. SEC, Secukinumab; ADA, Adalimumab; UPA, Upadacitinib; ACR, American College of Rheumatology; PsARC, Psoriatic Arthritis Response Criteria; VAS, Visual Analog Scale; LEI, Leeds Enthesitis Index; ASAS, Assessment of SpondyloArthritis International Society; PASI, Psoriasis Area and Severity Index; MDA, Minimal Disease Activity; HAQ, Health Assessment Questionnaire.

**Supplementary Table S3. Treatment effectiveness and outcomes at week 24.**

| **Variable** | **SEC** | **ADA** | **UPA** | ***P1*** | ***P2*** |
| --- | --- | --- | --- | --- | --- |
| **Peripheral arthritis** | n = 61 | n = 60 | n = 32 |  |  |
| ACR20 | 48 (78.69) | 48 (80.00) | 21 (65.62) | 0.89 | 0.27 |
| ACR50 | 45 (73.77) | 46 (76.67) | 20 (62.50) | 0.78 | 0.27 |
| ACR70 | 38 (62.30) | 41 (68.33) | 17 (53.12) | 0.54 | 0.27 |
| PsARC | 48 (78.69) | 49 (81.67) | 21 (65.62) | 0.78 | 0.15 |
| Change of pain VAS | -3.26 (3.12) | -4.17 (3.19) | -2.75 (2.75) | 0.11 | 0.07 |
| Change of LEI | -0.23 (1.07) | -0.40 (0.96) | -0.12 (1.01) | 0.31 | 0.18 |
|  |  |  |  |  |  |
| **Axial arthritis** | n = 49 | n = 42 | n = 23 |  |  |
| ASAS20 | 42 (85.71) | 34 (80.95) | 17 (73.91) | 0.45 | 0.50 |
| ASAS40 | 39 (79.59) | 33 (78.57) | 17 (73.91) | 0.86 | 0.78 |
| ASAS70 | 33 (67.35) | 28 (66.67) | 16 (69.57) | 0.73 | 0.80 |
| Change of back pain VAS | -1.65 (2.87) | -3.01 (3.51) | -2.17 (3.07) | **0.05** | 0.60 |
|  |  |  |  |  |  |
| **Skin** | n = 68 | n = 61 | n = 36 |  |  |
| PASI90 | 49 (72.06) | 29 (47.54) | 27 (75.00) | **0.004** | **0.04** |
| PASI75 | 56 (82.35) | 38 (62.30) | 29 (80.56) | **0.01** | 0.10 |
|  |  |  |  |  |  |
| **Global evaluation** | n = 69 | n = 62 | n = 36 |  |  |
| MDA | 53 (76.81) | 49 (79.03) | 28 (77.78) | 0.98 | 0.97 |
| Change of HAQ | -0.19 (0.58) | -0.24 (0.59) | -0.16 (0.44) | 0.52 | 0.95 |
| Change of disease activity-patients | -3.54 (3.25) | -3.80 (3.02) | -2.00 (3.97) | 0.78 | **0.02** |
| Change of disease activity-clinician | -3.22 (2.93) | -3.38 (2.82) | -2.25 (3.70) | 0.88 | 0.14 |
| Change of global VAS | -3.57 (2.85) | -3.77 (2.82) | -2.96 (2.87) | 0.76 | 0.27 |

Continuous variables are presented as mean (SD), and categorical variables are presented as counts (%). For binary outcomes, comparisons between treatment groups were performed using multivariable logistic regression models, adjusting for age, BMI, baseline PASI score, and history of prior biologic treatment. For continuous outcomes, multivariable linear regression models were used with the same covariate adjustments. *P1* represents the p-value for the comparison between SEC and ADA, while *P2* represents the p-value for the comparison between UPA and ADA. SEC, Secukinumab; ADA, Adalimumab; UPA, Upadacitinib; ACR, American College of Rheumatology; PsARC, Psoriatic Arthritis Response Criteria; VAS, Visual Analog Scale; LEI, Leeds Enthesitis Index; ASAS, Assessment of SpondyloArthritis International Society; PASI, Psoriasis Area and Severity Index; MDA, Minimal Disease Activity; HAQ, Health Assessment Questionnaire.
